# ESPO and Academy for Gerontology in Higher Education Section Symposium: Gerontological Education as an Interdisciplinary Pursuit

**DOI:** 10.1093/geroni/igaf122.407

**Published:** 2025-12-31

**Authors:** Abigail Stephan, Erta Cenko, Aimee Fox

## Abstract

As professionals across the globe recognize the critical need to support our aging population, training opportunities that allow students to experience the inherent interdisciplinarity of gerontology and geriatrics will be in high demand. Accordingly, this session explores innovative approaches for integrating gerontological concepts within educational experiences for students and early career professionals. We highlight collaborations among gerontology and geriatrics faculty and professionals from diverse fields to enhance student learning around aging. Presenters will share examples of successful partnerships and discuss challenges and opportunities in interdisciplinary gerontological education. In the first presentation, Cenko and Rani explore technology as a tool to incorporate gerontological concepts within health science education. Newsham and colleagues will then share about an innovative interdisciplinary collaboration between faculty and students exploring end-of-life and advance care planning. The third presentation centers the work of Carpenter and Aguilo, which examines an immersive visual arts program bringing together students and older artists. Stephan and colleagues will highlight a collaboration with landscape architecture and its impact on students’ perceptions of aging in our fourth presentation. Finally, Mancini will share insights from mentoring across the career spectrum. By showcasing how aging-related topics intersect with fields such as healthcare, social sciences, education, technology and engineering, and the arts, this session emphasizes the importance of interdisciplinary approaches in preparing students to address the complexities of an older society. Attendees will gain insights into practical strategies for expanding gerontological education across disciplines and fostering meaningful connections between aging studies and other fields.

